# Immunomodulatory Properties of Stem Cells in Periodontitis: Current Status and Future Prospective

**DOI:** 10.1155/2020/9836518

**Published:** 2020-07-08

**Authors:** Mengyuan Wang, Jiang Xie, Cong Wang, Dingping Zhong, Liang Xie, Hongzhi Fang

**Affiliations:** ^1^Department of Stomatology, The Third People's Hospital of Chengdu, The Affiliated Hospital of Southwest Jiaotong University, Chengdu, China 610000; ^2^The Third People's Hospital of Chengdu, The Affiliated Hospital of Southwest Jiaotong University, Chengdu, China 610000; ^3^State Key Laboratory of Oral Diseases, National Clinical Research Center for Oral Diseases, West China Hospital of Stomatology, Sichuan University, Chengdu, China 610041

## Abstract

Periodontitis is the sixth-most prevalent chronic inflammatory disease and gradually devastates tooth-supporting tissue. The complexity of periodontal tissue and the local inflammatory microenvironment poses great challenges to tissue repair. Recently, stem cells have been considered a promising strategy to treat tissue damage and inflammation because of their remarkable properties, including stemness, proliferation, migration, multilineage differentiation, and immunomodulation. Several varieties of stem cells can potentially be applied to periodontal regeneration, including dental mesenchymal stem cells (DMSCs), nonodontogenic stem cells, and induced pluripotent stem cells (iPSCs). In particular, these stem cells possess extensive immunoregulatory capacities. In periodontitis, these cells can exert anti-inflammatory effects and regenerate the periodontium. Stem cells derived from infected tissue possess typical stem cell characteristics with lower immunogenicity and immunosuppression. Several studies have demonstrated that these cells can also regenerate the periodontium. Furthermore, the interaction of stem cells with the surrounding infected microenvironment is critical to periodontal tissue repair. Though the immunomodulatory capabilities of stem cells are not entirely clarified, they show promise for therapeutic application in periodontitis. Here, we summarize the potential of stem cells for periodontium regeneration in periodontitis and focus on their characteristics and immunomodulatory properties as well as challenges and perspectives.

## 1. Background

Periodontitis is a chronic inflammatory condition that gradually devastates tooth-supporting tissue, which is comprised of the periodontal ligament (PDL), gingiva, and alveolar bone. The severe form of periodontitis, which impacts 743 million around the world, is the sixth-most prevalent chronic disease [1, 2]. Periodontitis is not only the main reason for tooth loss in adults but is also related to a variety of chronic diseases (i.e., diabetes, obesity, osteoporosis, arthritis, depression, cardiovascular disease, and Alzheimer's disease) [3–5].

Conventional therapies focus on utilizing natural and synthetic materials to fill defects of periodontal tissue, but these substitutes do not result in the actual restoration of the original physical structure and function of the tissue [6]. Due to the complexity of periodontal tissue, it is still a challenge to regenerate the periodontium. Tissue engineering approaches for regenerative dentistry consist of stem cells in the oral cavity, cytoskeleton, and growth factors. Stem cells exhibit highly promising therapeutic potential in periodontal regeneration owing to their self-renewal property and the plasticity of their potential to differentiate [7]. DMSCs, nonodontogenic stem cells, and iPSCs can be applied to periodontal tissue regeneration. Given the remarkable properties and versatility of stem cells, they are considered to be an efficient approach to regenerate periodontal tissue [8–10]. In addition, stem cells play a crucial role in immunosuppressive and anti-inflammatory functions [11]. In periodontitis, stem cells can be delivered to a site of infection and function as critical players to control inflammation and the immune response, achieving a regenerative process [12].

Here, we briefly summarize the potential of stem cells for periodontium regeneration, mainly focusing on their characteristics and immunomodulatory properties as well as the challenges and perspectives for their application.

## 2. Pathological Mechanism of Periodontitis

**Figure 1:**
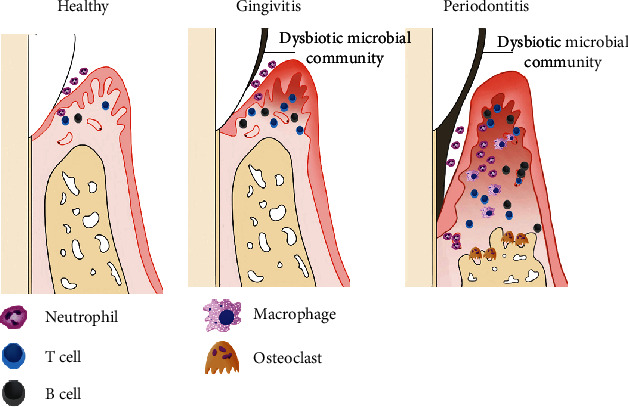
The pathological mechanism of periodontitis. Periodontal tissue homeostasis is dependent on the balance between the host immune defenses and microbial attacks. Once dysbiotic microbial communities subvert a susceptible host, the inflammatory dialog would be generated. Thus, dysbiotic microbiota act as a pathobiont which overactivate the inflammatory response, then trigger periodontal tissue breakdown associated with innate and adaptive immunoregulation, potentially resulting in resorption of supporting alveolar bone, even tooth loss and systemic complications.

Uncovering the mechanisms of inflammatory responses in periodontitis will facilitate the application of stem cells to treat this disease [13]. Periodontal tissue homeostasis is dependent on the balance between host immune defenses and microbial attacks [14]. Once the dysbiotic microbial community subverts a susceptible host, an inflammatory response is generated [15]. This process is mediated by the immune system of the host, which triggers the breakdown of tooth-supporting structures, resulting in the initiation of periodontitis (Figure 1).

### 2.1. Microbial Dysbiosis: The Causative Agent of Periodontitis

The dysbiotic microbial community consists of anaerobic bacterial genera, including *Proteobacteria*, *Firmicutes*, *Spirochaetes*, *Synergistetes*, and *Bacteroidetes* [16]. The subgingival microenvironment affords opportunities for the microbial community due to the enrichment of inflammatory mediators. The dysbiotic microbial community subverts host immune responses by enhancing their nutrient acquisition and evasion strategies in the inflammatory milieu. The dysbiotic oral microbiota display synergistic interactions that can cause reciprocal proteomic and transcriptomic responses to reinforce nutrient acquisition [17, 18]. The dysbiotic oral microbiota procure nutrients from destructive inflammatory tissue, including heme-containing composites and degraded collagen peptides [19]. These periodontal bacteria can improve their fitness by regulating the communication with the host immune response. For example, these bacteria escape neutrophil-mediated assault and protect themselves from complement. As a result, periodontal tissue breakdown is increased by neutrophil-mediated responses due to the inability of the neutrophil to control the dysbiotic microbial attack [20].

### 2.2. Host Susceptibility to Periodontitis

Host susceptibility to periodontitis not only governs the transition from microbial synergy to dysbiosis but also determines the development of inflammation and the progression of irreversible tissue destruction [21, 22]. The progression and severity of periodontitis rely on host-related factors, including immunoregulatory dysregulation, immunodeficiencies, systemic diseases related to periodontitis (such as diabetes, cardiovascular disease, obesity, osteoporosis, arthritis, depression, and Alzheimer's disease), risk factors affecting the host's immune system (such as smoking, stress, ageing, and microbial factors), and regenerative responses [23, 24]. Defects or dysregulation of the host immune response leads to an inability to suppress dysbiotic microbial communities and the resultant pathogenesis. The susceptible host immune response is subverted by dysbiotic microbiota, leading to the formation of a self-perpetuating pathogenic cycle [15].

### 2.3. Immune Response in Periodontitis

Once periodontitis is triggered by dysbiotic microbiota, the immune response in periodontitis changes from acute inflammation into the chronic condition and leads to the breakdown of the periodontium [25] (Figure 1).

Dysbiotic microbiota can reinforce their own tolerance to host immune responses by interacting with neutrophils and complement [14]. Neutrophils congregate in the gingival sulcus, while T cells, B cells, and monocytes are recruited. Neutrophils release elastase to degrade membrane proteins in some bacteria, which causes the breakdown of elastin and type IV collagen in the PDL and therefore disintegrates its attachment to the cementum and alveolar bone, leading to the formation of a periodontal pocket [26]. Neutrophils also secrete cytotoxic substances and degradative enzymes (i.e., reactive oxygen species and matrix metalloproteinases) that result in the inflammatory destruction of tissue [27]. In addition, neutrophils release the receptor activator of nuclear factor kappa-B ligand (RANKL), which is necessary for osteoclastogenesis and periodontal bone resorption [28]. Another major source of RANKL is via secretion from B cells and T cells in inflammatory lesions [29]. Specifically, neutrophils mediate the chemotactic recruitment of interleukin- (IL-) 17-mediated T helper 17 (Th17) cells through the expression of chemokine ligand (CCL) 2 and CCL20. Meanwhile, chemokine receptor (CCR) 2 and CCR6 are secreted by Th17 cells [30, 31]. Th17 cells are a subset of T cells that promote osteoclastogenesis and act as effective helpers of B cells [32]. The progression of periodontitis is characterized by inflammatory infiltration with large numbers of B cells and plasma cells accompanied by the increasing expression of immune complexes and complement fragments [33]. Specifically, B cells induce the conducive destruction of periodontal tissue which is due to matrix metalloproteinases and inflammatory cytokines secreted by B cells [34].

Macrophages remodel connective tissue by balancing matrix metalloproteinases and their tissue inhibitors. Macrophages also regulate bone homeostasis by mediating osteoblasts and osteoclasts. Moreover, this capacity of polymorphonuclear leukocytes and monocytes is achieved by the secretion of cytokines, including tumor necrosis factor *α* (TNF-*α*), adhesion molecules, IL-1*β*, and IL-6. These factors induce these cells to adhere to the endothelium and to increase the permeability of gingival capillaries and alveolar bone resorption [13, 35].

## 3. Characteristics, Immunological Properties, and Periodontal Regeneration Potential of Stem Cells

In this section, we review the characteristics as well as the immunological properties of stem cells, including DMSCs, nonodontogenic stem cells, and iPSCs. Specifically, we present stem cells as having potential efficacy for regenerating compromised tissues.

### 3.1. DMSCs

**Figure 2:**
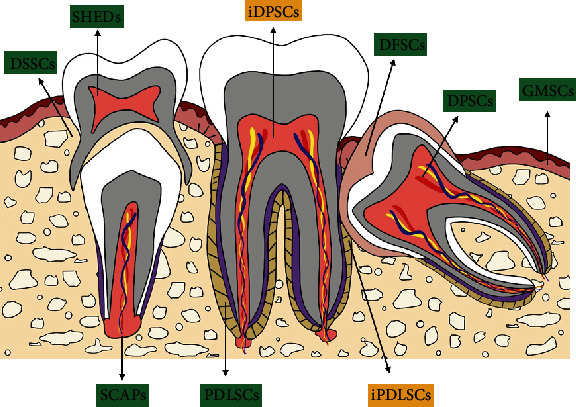
The different populations of dental mesenchymal stem cells and their distribution. PDLSCs: periodontal ligament stem cells; DFSCs: dental follicle stem cells; DPSCs: dental pulp-derived stem cells; SCAPs: stem cells from apical papilla; SHEDs: stem cells from exfoliated deciduous teeth; GMSCs: gingival mesenchymal stem cells; DSSCs: dental socket-derived stem cells; iPDLSCs: PDLSCs derived from infected tissue; iDPSCs: DPSCs derived from infected tissue.

DMSCs are composed of periodontal ligament stem cells (PDLSCs), dental follicle stem cells (DFSCs), dental pulp-derived stem cells (DPSCs), stem cells from apical papilla (SCAPs), stem cells from exfoliated deciduous teeth (SHEDs), gingival mesenchymal stem cells (GMSCs), and dental socket-derived stem cells (DSSCs) [36, 37] (Figure 2). DMSCs are multipotent stem cells with a self-renewal ability as well as multiple lineage differentiation potentials [38]. More importantly, DMSCs mediate the activity of various immune cells. The immunomodulatory potential of DMSCs mainly relies on inflammatory factors secreted by immune cells.

#### 3.1.1. PDLSCs

The PDL is a connective tissue that connects the tooth root to the surrounding alveolar bone. The PDL originates from the dental follicle and plays a critical role in sustaining tooth homeostasis and providing nutrition. PDLSCs were first derived and identified from adult third molars [39]. PDLSCs have the potential to generate PDLs, alveolar bone, cementum, blood vessels, and peripheral nerves [40]. In addition, these cells also have a self-renewal capacity and high proliferative potential [41]. PDLSCs expressed various types of MSC-related cluster of differentiation (CD) markers, including CD73, CD90, and CD105, and lack expression of hematopoietic markers, such as CD14, CD19, CD34, CD40, CD45, CD80, and CD86 [42–44]. Human PDLSCs also express antigens such as TRA-1-60, TRA-1-81, sex-determining region Y-box (Sox) 2, alkaline phosphatase (ALP), stage-specific embryonic antigen- (SSEA-) 1, SSEA-3, SSEA-4, and reduced expression 1 [6, 45].

**Figure 3:**
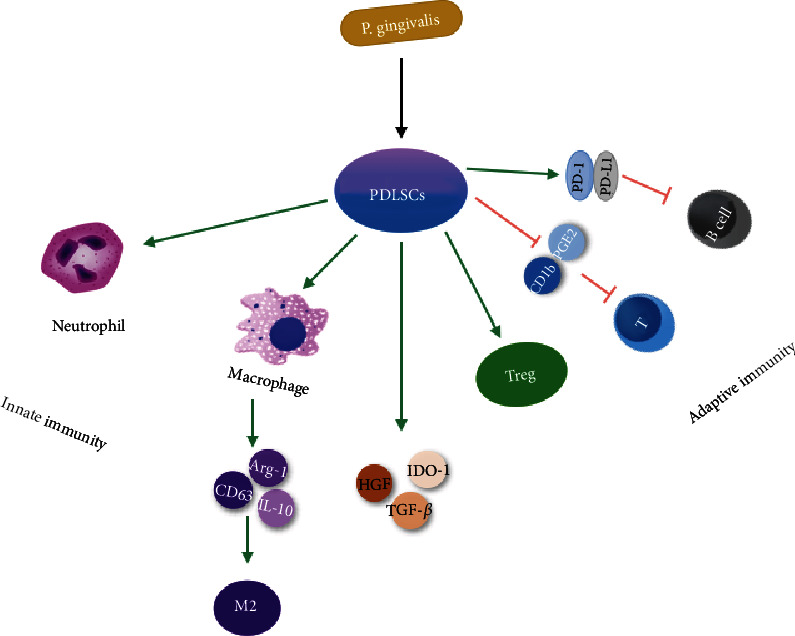
The immunological properties of PDLSCs linked with innate and adaptive immunity. PDLSCs possess immunoregulatory and anti-inflammatory capacities via both innate and adaptive immune responses.

Recently, PDLSCs were deemed to be a promising potential cell source for the repair of periodontal defects following periodontitis on account of their immunomodulatory properties (Figure 3). Activated human peripheral blood mononuclear cells (PBMCs) generate interferon- (IFN-) *γ*, which induces PDLSCs to secrete some soluble factors (i.e., transforming growth factor- (TGF-) *β*, indoleamine 2,3-dioxygenase-1 (IDO-1), and hepatocyte growth factor (HGF)) that, in turn, partially decrease the proliferation of PBMCs [46]. Upregulated proliferation and downregulated apoptosis of neutrophils constitute another innate immune response mediated by PDLSCs [47]. PDLSCs also greatly inhibit T cell proliferation by reducing the secretion of major histocompatibility complex glycoprotein1b (GP1b) and prostaglandin E2 (PGE2) from dendritic cells (DCs) [48]. Additionally, PDLSCs improve the activity and proliferation of anti-inflammatory Treg cells and suppress proinflammatory Th1/Th2/Th17 cells [49]. In addition to these cells, the mechanism of immunosuppression is mediated by PDLSCs through the inhibition of B cell proliferation, migration, and differentiation. These properties of PDLSCs are achieved via stimulating the expression of programmed cell death protein 1 (PD-1) and its ligand (PD-L1) [50]. PDLSCs enhance the polarization of the anti-inflammatory phenotype (M2 phenotype) by stimulating Arginase- (Arg-) 1, CD163, and IL-10 and inhibiting TNF-*α* [47].

Currently, both the animal experiments and clinical trials demonstrate that PDLSCs can regenerate periodontal defects. Studies have reported that delivery of PDLSCs to periodontal defects in rat models improves periodontal regeneration by generating PDLs, cementum-like tissue, and new bone without inflammation [51]. More importantly, PDLSCs achieve periodontal regeneration without adverse effects. In a miniature swine periodontitis model, transplantation of an allogeneic PDLSC sheet achieved the regeneration of the periodontium and cured periodontitis through immunosuppressive effects and low immunogenicity [52]. A clinical study exhibited that autologous PDLSC transplantation possessed the advantages of stability and effectiveness during the long-term follow-up of patients with periodontitis, suggesting that PDLSCs may be an innovative approach to treat periodontitis [46].

#### 3.1.2. DFSCs

DFSCs are responsible for periodontium formation by migrating around the tooth germ and differentiating into PDLs, osteoblasts, and cementoblasts [53]. Surface markers of DFSCs contain CD13, CD44, CD73, CD105, CD56, CD271, human leukocyte antigen- (HLA-) ABC, STRO-1, and NOTCH-1. Among them, STRO-1 and CD44 are common surface markers that are used to identify DFSCs [54].

The immunosuppressive properties of DFSCs are dependent on TLRs. In periodontitis, *P. gingivalis* and *F. nucleatums* activate the expression of TLR2 and TLR4 on the membrane of DFSCs and then trigger DFSCs to inhibit the proliferation of peripheral blood mononuclear cells (PBMCs) [55]. Moreover, DFSCs can upregulate the secretion of the anti-inflammatory cytokine IL-10 and simultaneously downregulate the levels of the proinflammatory cytokines IL-4, IL-8, and IFN-*γ*, thereby damaging bacterial adherence and internalization [56]. DFSCs exert anti-inflammatory effects and suppress bone degradation by mediating the phagocytic activity, chemotaxis, and NET formation of neutrophils and inducing macrophage polarization into the M2 phenotype [57].

Several animal experiments have proven that DFSCs possess the capacity to repair periodontal defects. In a canine model of periodontal defects, the potential of DFSCs to repair periodontal defects was proven via implantation of autologous DFSCs into defects, inducing the generation of new PDLs, alveolar bone, and cementum [36]. In another study, ectopic transplantation of DFSCs from human-impacted third molars into nude mice generated the cementum-PDL complex [58].

#### 3.1.3. DPSCs

DPSCs were the first characterized mesenchymal stem cells derived from the dental pulp in 2002. These cells possess the potential to differentiation into osteogenic, adipogenic, chondrogenic, and neural cells and show high expression of surface markers of MSCs [59].

DPSCs possess the ability to mediate both innate and adaptive immune responses by interacting with T cells, B cells, macrophages, and natural killer (NK) cells [60]. DPSCs exert anti-inflammatory and immunosuppressive effects by suppressing the proliferation of activated T cells as well as triggering apoptotic programmed cell death [61]. DPSCs also inhibit immunoglobulin production of B cells and IL-17 by increasing the secretion of IFN-*γ* [62]. The inhibitory effect of DPSCs on the proliferation of PBMCs is achieved by generating TGF and stimulating the mitogen-activated protein kinase (MAPK) signaling pathway [63]. DPSCs exert an anti-inflammatory function in two ways. On the one hand, DPSCs inhibit macrophages from secreting TNF-*α* by an IDO-dependent pathway. On the other hand, DPSCs initiate macrophage M2 polarization [64]. Induction of DPSC differentiation enhances the inhibitory effects of DPSCs on NK cell-mediated lysis and cytotoxicity.

In fact, Park and colleagues showed that DPSCs hardly repaired periodontal defects on account of their limited capacity to form a cementum-like structure, while PDLSCs regenerated the periodontium with new bone, cementum, and Sharpey's fibers [36]. As the function of DPSCs on pulp repair, there is little research about immunomodulatory properties of DPSCs in PDL tissue. Consequently, the present evidence indicates that DPSCs may not be an appropriate source for periodontal tissue engineering.

#### 3.1.4. SCAPs

SCAPs were first isolated from human apical papilla tissue of immature permanent teeth in 2006 [65]. Similar to other MSCs, SCAPs show a self-renewal capacity, high proliferative potential, and low immunogenicity as well as multilineage differentiation. STRO-1, CD24, and CD146 are widely expressed in SCAPs and are considered to be surface markers of SCAPs [66]. SCAPs can inhibit T cell proliferation by a mechanism independent of apoptosis [67]. It has also been reported that transplanting SCAPs into a periodontitis site significantly ameliorates the periodontitis parameters of periodontal tissue 12 weeks after transplantation [68]. All of these results suggest SCAPs may be a promising cell source for repairing the periodontium in regenerative dentistry.

#### 3.1.5. SHEDs

SHEDs were first characterized and isolated from the human dental pulp of exfoliated deciduous teeth by Miura. SHEDs show the ability to regenerate bone and dentin-like tissue, with a high osteoinductive ability and proliferation rate [62].

SHEDs show immunomodulatory characteristics via mediating T cell activation, maturation, and differentiation. Moreover, downregulation of Th17 cells and upregulation of regulatory T cells (Tregs) are additional immunosuppressive effects of SHEDs [69]. Furthermore, SHEDs can suppress DCs from secreting the inflammatory cytokines IL-2, IFN-*γ*, and TNF-*α* and can facilitate DCs to generate the anti-inflammatory factor IL-10 [70]. SHEDs induce polarization of bone marrow-derived macrophages towards M2 polarization, which contributes to the regeneration of the periodontium and anti-inflammatory effects in periodontal tissues [71].

In an experimental periodontitis model, delivery of SHEDs into periodontal tissues led to a reduction of cytokine expression, osteoclast differentiation, and gum bleeding as well as promoted the formation of new attachments of PDL and alveolar bone. These results suggest that SHEDs contribute to the improvement of periodontal regeneration and the decrease of periodontal tissue inflammation [72].

#### 3.1.6. GMSCs

The epithelium and connective tissue make up the human gingiva, which is considered to be an essential constituent of the periodontium that exerts remarkable effects on periodontal regeneration and immunity and is notable for its wound healing properties without scaring. GMSCs were derived and identified from the lamina propria of gingival tissue in 2009 [73]. Based on their remarkable self-renewal, multilineage differentiation, and regenerative abilities, GMSCs are expected to be a suitable cell source in periodontal tissue engineering.

Recently, the easy accessibility and prominent immunomodulatory properties of GMSCs have led to more attention on the use of cellular therapy [74]. GMSC-induced immunomodulation represents a promising perspective in therapy of periodontal tissue inflammation via interaction with inflammatory cells and cytokines [74]. GMSCs communicate with the inflammatory environment through the expression of TLRs 1, 2, 3, 4, 5, 6, 7, and 10, which affect the immunomodulatory properties of GMSCs [75]. Human GMSCs show the capacity to facilitate the polarization of macrophages to the M2 phenotype; meanwhile, they inhibit the activation of M1 macrophages by producing PGE2, IL-6, and IL-10 [76]. Furthermore, GMSCs significantly reduce the activation and maturation of DCs by a PGE2-related mechanism that suppresses the antigen presentation ability of DCs and weakens the inflammatory response [77]. Human GMSCs also reduce the proliferation and differentiation of Th1/Th2/Th17 cells. GMSCs have an inhibitory function on PHA-dependent T cell proliferation and activation by upregulating immunosuppressive factors, such as IDO and IL-10 [78].

A study reported that GMSCs mixed with an IL-1RA-hydrogel synthetic extracellular matrix, when delivered into a periodontitis model, led to an obvious improvement of regenerating PDLs, cementum, and alveolar bone [74]. In a dog model, the transplantation of GFP-labelled GMSCs into furcation defects obviously improved the regeneration of damaged periodontal tissues [79].

#### 3.1.7. DSSCs

Recent studies have shown that dental sockets can be a potential source for periodontal regeneration. DSSCs have the potential to form colonies and can differentiate into osteoblasts, adipocytes, and chondrocytes [80]. Compared with BMSCs, colony formation, proliferation, and motility of DSSCs are stronger. DSSCs can positively express surface markers of stem cells, such as CD44, CD90, and CD271, and lack expression of hematopoietic markers, such as CD34 and CD45 [81].

Nakajima et al. reported that the transplantation of autologous DSSCs mixed with *β*-TCP/PGA into one-wall periodontal defects regenerated a new periodontium with PDL-like and cementum-like tissues and alveolar bone [80]. There are few studies on DSSCs, so more preclinical investigations are required to clarify the roles of DSSCs in tissue regeneration and immune regulation.

### 3.2. Nonodontogenic Stem Cells

Nonodontogenic stem cells are composed of bone marrow stromal stem cells (BMSCs) and adipose tissue-derived stem cells (ASCs).

#### 3.2.1. BMSCs

BMSCs can differentiate into osteoblasts, chondrocytes, adipocytes, and muscle cells [82]. BMSCs are sorted by surface markers of octamer-binding transcription factor- (Oct-) 4, CD73, CD90, CD105, CD146, STRO-1, and Nanog and do not express HLA-DR, CD14, CD34, or CD45. BMSCs have the potential to regenerate periodontal defects by generating alveolar bone, Sharpey's fibers, and cementum [83]. BMSCs migrate into PDLs, alveolar bone, blood vessels, and cementum and differentiate into osteoblasts and fibroblasts after local or systematic transplantation [84].

Aside from regenerating destroyed tissues in periodontitis, BMSCs also play a crucial role in anti-inflammation and immunosuppressive function [85]. BMSCs mediate the survival and proliferation of T lymphocytes for the regulation of immunomodulation [86]. BMSCs inhibit inflammatory cytokines, including IL-1 and TNF-*α*, which indicates that the use of BMSCs for the treatment of chronic periodontitis might be feasible [85]. In a clinical study, the combined use of autologous BMSCs and platelet-rich plasma to treat periodontal defects shows obvious tissue regeneration effects [87]. Although a significant improvement in periodontal parameters has been observed, more clinical research is needed to reveal the function of BMSCs and their ability to regulate inflammation and immunity to better target them for the treatment of periodontitis.

#### 3.2.2. ASCs

The characteristics of ASCs are similar to those of BMSCs, such as expression of the markers STRO-1, CD29, CD44, CD71, CD90, and CD105 and the lack of expression of hematopoietic cell markers CD31, CD34, and CD45 [88]. ASCs can differentiate into osteoblast, adipocytes, chondrocytes, myogenic, and neurogenic cells. Compared to BMSCs, ASCs are superior because of their easier harvesting process and because of their fewer notable donor site complications [89]. More importantly, ASCs mixed with cytokines TNF-*α*, IFN-*γ*, and IL-6 promote the expression of immune suppressive factors, including GBP4 and IL-1RA [90].

Preclinical studies have demonstrated that ASCs are potential candidate cells for the regeneration of periodontal destruction. ASCs can secrete growth factors, such as insulin-like growth factor binding protein-6, which facilitates the differentiation of ASCs into the periodontium [91]. Allogeneic ASCs were transplanted in a microminipig model of periodontal tissue defects and led to the generation of new PDL-like fibers, alveolar bone, and the cementum in defect sites [90].

### 3.3. Induced Pluripotent Stem Cells (iPSCs)

The formation of iPSCs can be achieved by reprogramming somatic cells with the transcriptional markers Oct4, Sox2, Krüppel-like factor 4, and Myc [92]. iPSCs express special pluripotent markers, including TRA160, TRA180, MSC-heat shock protein 90, CD73, CD90, CD105, CD146, and CD106 [93]. iPSCs are pluripotent stem cells with the potential to generate iPSC-derived MSCs (iPSC-MSCs) and to differentiate into multilineage cells [94]. As a promising candidate, iPSCs not only have the potential to regenerate bone, cartilage, brain, heart, and liver tissue but also can be applied for inflammatory tissue regeneration in periodontitis [95]. Stem cells from dental tissue, including PDLs, buccal mucosa fibroblasts, gingival, apical papilla, and the dental pulp, have advantages for the generation of iPSCs [96].

Moreover, iPSC-MSCs can inhibit Th1/Th2/Th17 cells and upregulate the expression of Treg cells, suppressing the production of leukocytes and alveolar bone resorption [97, 98]. iPSCs are a potential cell source for the clinical prevention and treatment of periodontitis. Duan et al. showed that transplantation of iPSCs to a scaffold with enamel-derived factors significantly increased PDL, alveolar bone, and cementum formation in a mouse periodontal defect model compared with iPSC-empty groups [99]. Another study reported that iPSCs could inhibit inflammation and decrease alveolar bone resorption in a rat model of periodontitis. In addition, Hynes et al. stated that iPSC-MSCs could repair periodontal tissue defects and control inflammation while lessening alveolar bone destruction [100].

## 4. Interaction of Stem Cells with the Inflammatory Milieu of the Periodontium

What happens to the stem cells in periodontitis and how they interact with periodontal inflammation are crucial for the application of stem cells into periodontal regeneration [101]. For instance, the interaction of stem cells and immune cells in the inflammatory milieu may be completely different from that in a healthy state with altered regenerative processes and immunomodulatory properties [102]. Thus, it is essential to understand the properties of stem cells derived from inflammatory tissue as well as the inflammatory responses and immunomodulation properties of stem cells in an inflamed microenvironment.

### 4.1. Stem Cells from Inflammatory Tissue

Inflamed stem cells exhibit characteristics including maintenance of stemness, formation of colonies, a higher proliferation rate, multilineage differentiation potential, and lower immunogenicity and immunosuppression [102].

In this section, we introduce the properties and immunoregulation of DMSCs in inflammatory periodontal sites. Among stem cells, DMSCs derived from infected tissue possess the significant advantages of being easily accessible and having fewer ethical complications [103]. Compared with other DMSCs, PDLSCs are considered to be an ideal cell source for periodontal regeneration [104], but there are still problems with obtaining enough PDLSCs from healthy donor sources. PDLSCs derived from inflamed periodontal sites are considered inflammatory periodontal ligament stem cells (iPDLSCs). Compared to PDLSCs, iPDLSCs have higher proliferative and migratory capacities. However, iPDLSCs exhibit lower osteogenic differentiation because of alterations of the osteogenesis-related signaling pathway, such as the Wnt/*β*-catenin, noncanonical Wnt/Ca^2+^, p38-MAPK, and NF-*κ*B signaling pathways [105, 106]. More importantly, iPDLSCs also have reduced immunosuppressive properties and less efficiently suppress T cell proliferation, PBMC proliferation, and Th17 differentiation in contrast to cells from healthy tissue [107]. High levels of IFN-*γ*, TNF-*α*, IL-2, and IDO and low expression of IL-10 are also characteristic of iPDLSCs [108]. A study reporting on the transplantation of collagen sponges combined with iPDLSCs isolated from inflamed human periodontal tissue into immunodeficient nude rats led to the formation of new PDL-like tissue, bone, and collagen fibers. Although complete regeneration was not achieved, the repair effect of iPDLSCs on periodontal defects was similar to that of PDLSCs from healthy periodontal tissues [51]. Compared with normal DPSCs, DPSCs derived from infected tissue (iDPSCs) show similar surface marker expression, proliferation properties, and multilineage differentiation potential [109, 110]. DPSCs derived from infected human tissue were layered onto *β*-tricalcium phosphate and grafted into periodontal defects in the root furcation. The outcome revealed new formation of alveolar bone [111]. These results have important implications for achieving periodontium regeneration with DMSCs obtained from inflammatory tissues in the future [112]. It may be a promising strategy to cultivate or even genetically modify DMSCs obtained from infected tissue, avoiding the destruction of the healthy periodontium while implanting DMSCs into inflamed periodontium tissue to achieve regeneration [113, 114].

However, there are still various issues that should be taken into account before translational application. For example, the source of inflamed stem cells, the inflammatory status, and the experimental design are confounding variables that will affect the quality and quantity of stem cells [115]. Furthermore, the inclusion criteria as well as the procedure for the isolation and transplantation of the inflamed stem cells should be established and standardized to further explore and verify the long-term effects of inflamed stem cells via *in vivo* and *in vitro* experiments [57, 116, 117].

### 4.2. Effect of the Infected Microenvironment on Stem Cells

The interaction of stem cells with the surrounding infected microenvironment could affect the mechanism of periodontal tissue repair and the regeneration outcome [102]. Transplantation of stem cells into periodontal defects is usually performed in an inflamed periodontal milieu, and the immunomodulatory capacity of stem cells is determined by diverse inflammatory cytokines. Therefore, understanding the effect of inflammatory cytokines on stem cells is critical to optimize and implement stem cell-mediated clinical approaches [118, 119].

Various inflammatory cytokines can specifically mediate the immunomodulatory activity of stem cells [120]. Among various inflammatory mediators, TNF-*α*, IL-1*β*, IL-6, and IFN-*γ* are the most effective proinflammatory cytokines during periodontitis [121]. The proinflammatory cytokines TNF-*α*, IL-1*α*, IL-1*β*, and IFN-*γ* exert critical effects by mitigating the immunosuppressive capacities of stem cells [122]. Low levels of IFN-*γ* improve antigen-presenting functions of stem cells and thus reduce their lysis. In contrast, high levels would reverse their antigen-presenting functions and show the opposite effect [123, 124].

Several studies have demonstrated the effects of an infected microenvironment on DMSCs. For example, *P. gingivalis*-LPS significantly enhanced cellular proliferation of DMSCs [125]. In addition, coculturing PDLSCs with IL-1*β*/TNF-*α* could enhance the proliferation rate of PDLSCs [126]. The surface markers of DMSCs, such as PDLSCs and GMSCs, do not change within the IL-1*β*/TNF-*α*-inflamed microenvironment. However, the effect may be compromised or may even lead to stem cell apoptosis when the IL-1*β*/TNF-*α* stimulus surpasses a certain level. The differentiation potential of DMSCs could be mediated by proinflammatory cytokines and microbial pathogens [127]. Specifically, *P. gingivalis*-LPS and *E.coli*-LPS inhibit PDLSCs' osteoblastic differentiation [125, 128]. IL-1*β*/TNF-*α* are responsible for reducing the osteogenesis of PDLSCs by stimulating the canonical Wnt/*β*-catenin pathway and inhibiting the noncanonical Wnt/Ca^2+^ pathway in the local periodontal milieu [106].

Other stem cells also exert important effects on the infected microenvironment. An increasing number of studies have shown that IFN-*γ* is required for BMSCs to exert their immunosuppressive effect on T lymphocyte proliferation. Additionally, both LPS and IFN-*γ* can induce the secretion of functional IDO and IL-10 by BMSCs [129]. LPS-induced proliferation of PBMCs could be inhibited by BMSCs [73]. Transplantation of BMSCs into LPS-stimulated models could inhibit the production of inflammatory cytokines and ameliorate inflammatory tissue destruction [130]. The interaction of ASCs with the inflammatory microenvironment is necessary to achieve tissue regeneration. In response to inflammatory cytokines, ASCs facilitate the anti-inflammatory and immunosuppressive potential through the induction of polarization of macrophages to the M2 phenotype [131]. When stimulated with TNF-*α* and IFN-*γ*, ASCs significantly increased their immunomodulatory capacities [132].

## 5. Conclusion, Future Clinical Application, and Challenges

To date, application of extracellular matrix scaffolds, bone grafts, and growth factors achieve only limited regeneration of intrabony defects [133]. In the past few years, developing data have indicated that stem cells have great potential in periodontitis due to the positive inflammatory-regenerative effects of these cells in the inflamed microenvironment [36, 134]. These stem cells have remarkable properties and versatility due to their stemness, proliferation, migration, and multilineage differentiation abilities and their immunosuppressive and anti-inflammatory functions in a local inflamed microenvironment [118, 135, 136].

The immunoregulatory effects of stem cells make them a promising therapy for periodontitis. Although several reports have indicated that the stem cells mentioned above can be delivered into infectious sites and function as critical players in the control of inflammation and the regulation of immune responses to achieve regeneration in models of periodontitis, the immunomodulatory capabilities of these cells have not entirely been elucidated [137, 138]. Moreover, evidence of stem cell-mediated immunomodulation is limited both *in vitro* and *in vivo*. It is very complicated to recreate extremely polluted surroundings in animal models because human periodontal lesions are filled with granulation tissue, calculus, pathogenic biofilms, and plaque [139]. Furthermore, there are various differences in the mechanisms of stem cell-mediated immunomodulation between humans and animals [140]. The quality and quantity of stem cells can be modulated by numerous factors, including the sources of stem cells and the experimental design [141]. The inclusion of subjects and procedures for the isolation and transplantation of stem cells can influence the outcome of regeneration and immunomodulation.

Therefore, inclusion criteria as well as a standard procedure for the isolation and transplantation of the stem cells should be established to further explore the long-term effects of stem cells. Standard animal models of periodontitis should be constructed to mimic human periodontal lesions. Selection of suitable biomaterial scaffolds and the appropriate combination with growth factors for stem cells may improve their periodontal regeneration and immunosuppression functions. More importantly, preclinical studies and clinical trials are critical to understand the mechanism of stem cell-mediated immunomodulation in the inflammatory milieu to pave the way for applying stem cells to periodontal tissue engineering (Table 1).

## Figures and Tables

**Table 1:** The characteristic of different stem cells could be potentially applied to periodontal regeneration.

Stem cell	Multipotent differentiation	Immunomodulatory properties	Clinical trails
*DMSCs*			
PDLSCs	Osteoblast, adipocytes, chondrocytes, cementoblast, and neurogenic cells	Inhibition of PBMCs, T cells, B cells, promotion of Treg cells, neutrophils, and M2 phenotype macrophage	NCT01357785 NCT01082822

DFSCs	Osteoblast, adipocytes, chondrocytes, cementoblast, neurogenic cells, cardiomyocyte, and dentin-like cell	Inhibition of PBMCs, promotion of Treg cells, neutrophils, and M2 phenotype macrophage	

DPSCs	Osteoblast, adipocytes, odontoblast, neurogenic cells, cardiomyocyte, and hepatocyte	Inhibition of PBMCs, T cells, B cells, and NK cells; promotion of Treg cells, neutrophils, and M2 phenotype macrophage	NCT03386877 NCT02523651

SCAPs	Osteoblast, adipocytes, odontoblast, neurogenic cells, and hepatocyte	Low immunogenicity; inhibition of T cells	

SHEDs	Osteoblast, adipocytes, chondrocytes, and neurogenic cells	Inhibition of Th17 cells; promotion of Treg cells and M2 phenotype macrophage	

GMSCs	Osteoblast, adipocytes, chondrocytes, and neurogenic cells	Inhibition of M1 macrophages, Th1/Th2/Th17 cells, and DCs; promotion of Treg cells and M2 phenotype macrophage	NCT03137979

DSSCs	Osteoblast, adipocytes, and chondrocytes	No report	

*Nonodontogenic stem cells*			
BMSCs	Osteoblast, adipocytes, and chondrocytes	Inhibition of T lymphocyte survival and proliferation; secretion of IL-1 and TNF-*α*	NCT02449005

ASCs	Osteoblast, adipocytes, chondrocytes, myogenic cells, and neurogenic cells	Promotion of immune suppressive factors GBP4 and IL-1RA	NCT04270006

*iPSCs*			
iPSCs	Osteoblast, adipocytes, chondrocytes, myogenic cells, neurogenic cells, cementoblast, cardiomyocyte, and dentin-like cell	Inhibition of Th1/Th2/Th17 cells; promotion of Treg cells	

The clinical trial data have been extracted from https://clinicaltrials.gov/.

## Data Availability

The human clinical trials data included in this review are available at https://clinicaltrials.gov/.

## Abbreviations

DMSCs: Dental mesenchymal stem cells
iPSCs: Induced pluripotent stem cells
iDMSCs: DMSCs derived from infected tissue
PDL: Periodontal ligament
TLRs: Toll-like receptors
RANKL: Receptor activator of nuclear factor kappa-B ligand
IL: Interleukin
Th17: T helper 17
CCL: Chemokine ligand
CCR: Chemokine receptor
TNF-*α*: Tumor necrosis factor *α*
PDLSCs: Periodontal ligament stem cells
DFSCs: Dental follicle stem cells
DPSCs: Dental pulp-derived stem cells
SCAPs: Stem cells from apical papilla
SHED: Stem cells from exfoliated deciduous teeth
GMSCs: Gingival mesenchymal stem cells
DSSCs: Dental socket-derived stem cells
Sox: Sex-determining region Y-box
ALP: Alkaline phosphatase
SSEA: Stage-specific embryonic antigen
CD: Cluster of differentiation
PGE2: Prostaglandin E2
PBMCs: Peripheral blood mononuclear cells
IFN: Interferon
TGF-*β*: Transforming growth factor-*β*
IDO-1: Indoleamine 2,3-dioxygenase-1
HGF: Hepatocyte growth factor
PD-1: Programmed cell death protein 1
PD-L1: Programmed cell death protein ligand 1
Arg: Arginase
HLA: Human leukocyte antigen
NK cells: Natural killer cells
MAPK: Mitogen-activated protein kinase
Tregs: Regulatory T cells
DCs: Dendritic cells
BMSCs: Bone marrow stromal stem cells
ASCs: Adipose tissue-derived stem cells
Oct: Octamer-binding transcription factor
HLA: Human leukocyte antigen
iPDLSCs: Inflammatory periodontal ligament stem cells.
